# Adiponectin Reduces Embryonic Loss Rate and Ameliorates Trophoblast Apoptosis in Early Pregnancy of Mice with Polycystic Ovary Syndrome by Affecting the AMPK/PI3K/Akt/FoxO3a Signaling Pathway

**DOI:** 10.1007/s43032-020-00237-z

**Published:** 2020-06-25

**Authors:** Wenqian Zhang, Meng Zuo, Juan Lu, Yuxia Wang

**Affiliations:** Department of Reproductive Medicine, The First Affiliated Hospital, Jinan University, 601 West Huangpu Avenue, Guangzhou, 510000 Guangdong People’s Republic Of China

**Keywords:** Polycystic ovarian syndrome, Adiponectin, Trophoblast cells, AMPK, PI3K, AKT

## Abstract

Reports in recent years have suggested that adiponectin (APN) improves insulin resistance and inhibits apoptosis by activating the AMP-activated protein kinase (AMPK) pathway and the PI3K/Akt signaling pathway after binding to its receptor. This study aims to explore the mechanism by which APN reduces embryo loss rate and trophoblast apoptosis in early pregnancy of mice with polycystic ovary syndrome (PCOS). PCOS mice were subcutaneously injected with APN (10 μg mg kg^−1^ day^−1^) on 11 consecutive days from the 3rd day of pregnancy onwards to observe the change of the embryo loss rate of PCOS mice induced by APN. Quantitative real-time PCR and Western blot were used to determine the relative expressions of mRNA and the proteins AMPK, PI3K, and Akt in mouse uterine tissue. At the same time, primary cultured mouse villous trophoblast cells were used to further explore the underlying mechanisms in vitro. APN significantly reduces the pregnancy loss rate of PCOS mice. At the same time, APN increases phosphorylation and mRNA expression levels of AMPK, PI3K, and Akt in PCOS mouse uterine tissue. In addition, trophoblast cells of model mice were treated with APN and inhibitors, and APN was found to reduce trophoblast cell apoptosis by affecting the phosphorylation levels of AMPK, PI3K, Akt, and FoxO3a proteins. APN reduces the embryo loss rate and ameliorates trophoblast apoptosis in PCOS mice by affecting the AMPK/PI3K/AKT/FoxO3a signaling pathway.

## Introduction

Polycystic ovarian syndrome (PCOS) is one of the most common endocrine and metabolic disorders in women of childbearing age. PCOS has an average incidence of 6–20% [1, 2], and obesity may establish a crucial barrier for effective fertility treatment in females who have PCOS. Obese women with PCOS had a lower rate of clinical pregnancy and a higher rate of abortions [3]. The etiology of PCOS is not yet fully understood. As researchers around the world gain a better understanding of PCOS, the awareness of the multisystem characteristics of this disease is increasing [4, 5].The syndrome can cause a range of health problems, including hyperandrogenemia (HA), insulin resistance (IR), ovulatory dysfunction, infertility, miscarriage, hyperlipidemia, cardiovascular disease, endometrial dysfunction, and obesity [6]. Therefore, PCOS is no longer simply considered an ovarian disease [7].

APN has anti-inflammatory, antioxidant, and anti-atherosclerosis properties and can also increase the insulin sensitivity and inhibit apoptosis. Both animal experiments and human in vitro fertilization (IVF) have confirmed the important role of APN in promoting follicular maturation and early embryonic development [8, 9]. In PCOS pregnancy patients and recurrent spontaneous abortion (RSA) patients, the plasma APN level was significantly lower than in normal pregnancy patients [10, 11].

APN regulates glucose and fatty acid metabolisms by binding to its receptor and activating the AMP-activated protein kinase (AMPK) pathway [12]. Compared with non-PCOS patients, AMPKα phosphorylation of the endometrium in PCOS patients is reduced, and the regulation of cell metabolism and growth is significantly reduced [13]. APN binding with its receptor can also play a role in improving insulin resistance, promoting trophoblast cell proliferation, and regulating the activity of apoptosis–profilin by activating the PI3K/Akt signaling pathway [14, 15]. FoxO3a protein is a direct target of the PI3K/Akt signaling pathway [16]. At the same time, FoxO3a plays an important role in the development of embryos, maturation of follicles, apoptosis of cells, tissue glucose homeostasis, and other biological development processes [17]. This suggests that APN probably activates the PI3K/Akt pathway by activating AMPK, thus enhancing the phosphorylation of FoxO3a. At the same time, it enhances the insulin signal transduction of fat cells, improves the insulin resistance and early embryonic development of PCOS patients, inhibits the apoptosis of trophoblast cells, and thus reduces the embryo loss rate in early pregnancy.

Appropriate expression of APN has been shown to maintain a normal pregnancy. Furthermore, APN directly regulates reproductive processes and implantation processes and promotes the synthesis and invasion of trophoblast cells in early pregnancy [18]. However, the underlying molecular mechanism has not been elucidated yet. In this study, we found that exogenous APN reduces the embryo loss rate and inhibits trophoblast apoptosis in PCOS mice by affecting the AMPK/PI3K/Akt/FoxO3a pathway. We demonstrated at the molecular level that the APN pathway ameliorates trophoblast cell apoptosis of PCOS pregnant mice, thus providing a new theoretical basis for the etiology of the high abortion rate caused by PCOS and proposing a new direction of thinking for its clinical treatment.

## Materials and Methods

### Animals

All studies involving animals are reported in accordance with the McGrath JC et al. [19]. All animal experiments were in compliance with the Ethical Committee of Laboratory Animals of Jinan University (Approval No.: I ACUC–20190125–08). In total, 66 female C57BL/6 mice and 25 male C57BL/6 mice (4 weeks old) weighing 12.5 g were obtained from Pengyue Experimental Animal Co., Ltd. with a Certificate of Quality No. SCXK (LU) 2014-0007 (Jinan, China). The animals were housed at 20 ± 5 °C, 43–47% relative humidity, and 12 h light–dark cycle. Before the experiment, the mice were kept for 1 week for acclimatization to the conditions. The mice were weighed daily during adaptive feeding and modeling. The standard laboratory diet and water were provided ad libitum.

### PCOS Mouse Model Development

We first randomly selected a female mouse to be included in the PCOS model group for pre-experimental use and then randomly divided the remaining 65 female mice into 5 groups with 13 females in each group. A group was randomly selected from the five groups as the control group. The other four groups (total 52 mice), along with the mouse that was randomly selected before (total 53 mice), were used for PCOS modeling. As a result, 66 female mice were randomly divided into model group (*n* = 53) and control group (*n* = 13). The model mice were given a 60% high-fat diet. They were subcutaneously injected with dehydroepiandrosterone (DHEA; Coolober technology Co., Ltd., Beijing, China; 0.6 mg kg^−1^ day^−1^, dissolved in 0.2 mL of soy oil) for 21 consecutive days. Mice in the control group were given a normal diet and subcutaneously injected with the same dose of 0.9% sodium chloride solution. On the first day after modeling, three mice in each group were randomly selected, blood was extracted after anesthesia, and serum-free testosterone was detected using Mouse testosterone ELISA Kit (CUSABIO, Wuhan, China). After the animals were killed, the ovaries were rapidly fixed in 4% paraformaldehyde solution. After being embedded in paraffin, they were cut into 4-μm-thick sections and stained with hematoxylin-eosin (H&E).

### Experimental Protocol

After successful modeling, 60 female mice were first intraperitoneally injected with pregnant horse serum gonadotropin (PMSG; 10 IU; ProSpec Bio, East Brunswick, NJ, Israel) and 48 h later with human chorionic gonadotropin (HCG; 10 IU; LIVZON, Guangdong, China). Then, female mice were mated with sexually mature male mice (8 w, 25 g). Mice with vaginal plug were recorded at day 0 of pregnancy. Except for the cell experimental mice, all the other mice were treated from the third day of pregnancy onwards.

Adiponectin (APN; gAcrp30/adiponectin, murine recombinant, BioVision, Milpitas, CA, USA) was dissolved in 1 mL Tris solution (5 mM, pH 7.6) to obtain an APN solution of a concentration of 0.1 mg/mL. The animals were randomly divided into four groups: cell experimental group (*n* = 10; without sodium chloride injection and APN), control group (*n* = 10; 0.9% sodium chloride injection, 10 μg mg kg^−1^ day^−1^, s.c., without APN, 11 days), PCOS group (*n* = 20; 0.9% sodium chloride injection, 10 μg mg kg^−1^ day^−1^, s.c., without APN, 11 days), and APN + PCOS group (*n* = 20, APN, 10 μg mg kg^−1^ day^−1^, s.c., 11 days).

### Tissue Preparation

On the 13th day of gestation, all the mice except for those of the cell experimental group were anesthetized and killed. The uterus was removed by laparotomy, and pregnancy was recorded. Embryo size, bleeding, necrosis, and absorption of pregnant mice were observed and compared with normal embryos. The number of absorbed embryos and the number of viable embryos were recorded. The embryo absorption rate is calculated according to the formula: embryo absorption rate = number of absorbed embryos / (number of absorbed embryos + number of survived embryos) × 100%.

### Cell Culture and Immunofluorescence

Trophoblast cells were obtained from PCOS model mice at day 19 of gestation. Pregnant mice in the cell experimental group were anesthetized and killed. Mice were immersed for 3 min in 75% ethanol for disinfection. The abdominal cavity of the mice was opened, and the uterus was cut lengthwise and aseptically dissected to remove decidual tissues and fetal membranes on a super clean bench. Chorionic tissues of the mice were separated under a microscope, washed repeatedly, and then cut into pieces of 1 ~ 3 mm^3^ with eye scissors. All operations were performed on ice to maintain the cellular activity. The cells were incubated with 0.25% trypsin for 30 min at 37 °C. Digestion was terminated by the addition of DMEM/F12 medium (GIBCO BRL, Grand Island, NY, USA) containing 10% fetal bovine serum (FBS, GIBCO BRL, Grand Island, NY, USA). Cells and tissues were mixed thoroughly by pipetting, filtered through a sterile 100-mm nylon mesh, and centrifuged for 10 min at 1000 r/min. The supernatant was discarded, and the pellet was suspended in the DMEM/F12 culture solution without FBS. The undigested tissue was digested twice, and the cell suspension was collected three times. The cell suspension was added into a 15-mL centrifuge tube containing 60% and 35% Percoll (Solarbio Science & Technology Co., Ltd., Beijing, China) to achieve a volume ratio of 1:1:1, resulting in the formation of three liquid surface layers. The tube was centrifuged for 20 min at 1500 r/min, and the separation layer was extracted from Percoll and centrifuged after PBS addition. The supernatant was discarded, and the previous steps were repeated. Finally, the cells were suspended in DMEM/F12 containing 20% FBS, and the density of cells was adjusted to 1 × 10^8^/L. Then, the cells were inoculated in a culture bottle coated with matrix glue. After cell isolation, the cells were cultured for about 1 week.

A week later, after the cells had grown on coverslips, the medium was removed, and the cells were once washed with PBS. Subsequently, the cells were fixed in 4% paraformaldehyde (PFA) for 30 min at 4 °C, washed three times with PBS for 5 min each time, and kept overnight at 4 °C in PBS. Then, 50 μL blocking buffer (1:1 mixture of 0.5% Triton X–100 and PBS containing 10% goat serum (Solarbio Science & Technology Co., Ltd., Beijing, China) was used to form closed droplets on the waterproof membrane and cover the slides for 2 h.

The cells were then incubated with anti-cytokeratin 18 antibody (CK18, 1:100 dilution, Abcam, Cambridge, MA, USA) overnight at 4 °C. CK18 is a well-identified specific cytoskeletal protein of trophoblast cells [20]. After washing with PBS, Alexa Fluor 488-conjugated Affinipure Goat Anti-Rabbit lgG (H + L) secondary antibody (Proteintech Group, Inc., Chicago, USA, 1:500 dilution) was applied for 2 h at room temperature. The cells were stained and then photographed under a fluorescence microscope (Olympus, Tokyo, Japan).

### MTT Assays

The cells were seeded into 96-well plates at a density of 8 × 10^3^ cells per well in complete culture medium. Following growth and attachment, APN of different concentrations was added, and the cell viability was assayed after 48 h. Therefore, 10 μL MTT (5 mg/mL; Solarbio Science & Technology Co., Ltd., Beijing, China) was added into each well, and the cells were incubated for 4 h. Then, 100 μL of formazan (iCell, Shanghai, China) solution was added into each well. After mixing and incubation in the incubator, a solution was obtained. The absorbance was measured in each well at 570 nm, and the increase in absorbance was recorded.

### Quantitative Real-Time PCR

Total RNA was extracted from the uterine tissue of mice using TRIzol reagent (Invitrogen, Carlsbad, CA, USA) to synthesize first-strand cDNAs by using the EasyScript TM First-strand cDNA Synthesis SuperMix (TransGen Biotech, Beijing, China). Total cDNA was determined by quantitative real-time PCR (qRT-PCR) on a 7500 real-time PCR system (Applied Biosystems, Thermo Fisher Scientific, Waltham, MA, USA). The qRT-PCR was performed using the ImProm-II™ Reverse Transcription System for analysis (Promega, Madison, WI, USA). Sangon Biotech (Shanghai, China) provided the specific primers for amplifying the specific genes. Table 1 shows the primer sequences that were used in this study. Expression levels were normalized to GAPDH, determined by the 2^-ΔΔCt^ method, and given as ratio compared with those of the control.

**Table 1:** Primer sequences for RT-PCR

Primer name	Nucleotide sequence (5′-3′)
AKT - forward	CAAGGCCCAACACCTTTATC
AKT - reverse	ACGATGACCTCCTTCTTGAG
AMPK - forward	TGTAGAGCAATCAAGCAGTT
AMPK - reverse	TCCTTTGGCAAGATCGATAG
PI3K - forward	GGGCAGTTAAGAAGCACAATG
PI3K - reverse	GCAGGAGAGTCTTTCCAATG
GAPDH - forward	GGCCTCCAAGGAGTAAGAAA
GAPDH - reverse	GCCCCTCCTGTTATTATGG

### Western Blot

Protein was extracted from uterine tissues and trophoblast cells of PCOS mice. Total protein concentrations were measured using a BCA protein assay kit (Solarbio Science & Technology Co., Ltd., Beijing, China). Equal amounts of protein (15 μg) were resolved by SDS-PAGE and then electrophoretically transferred to PVDF membranes. Subsequently, the membrane was blocked with 5% BSA (Solarbio Science & Technology Co., Ltd., Beijing, China) for 2 h and probed with different primary antibodies at 4 °C overnight. The main primary antibodies were phospho-AMPKα (Thr172; Cell Signaling Technology, Danvers, MA, USA), phospho-PI3 kinase p85 (Tyr458)/p55(Tyr199) antibody (Cell Signaling Technology, Danvers, MA, USA), phospho-Akt (Ser 473) antibody (Cell Signaling Technology, Danvers, MA, USA), phospho-Fox03a (Ser 253) antibody (Cell Signaling Technology, Danvers, MA, USA), caspase-3 antibody (Cell Signaling Technology, Danvers, MA, USA), and Fox03a antibody (Abcam, Cambridge, MA, UK). After washing thoroughly three times with TBST for 5 min each time, HRP-linked secondary antibody (Cell Signaling Technology, Danvers, MA, USA) was used to detect the primary antibodies, followed by an additional 1-h incubation at room temperature. At last, bands were visualized using the Amersham Imager 600 (General Electric Company, Boston, MA, USA), and band intensities were analyzed using the software Image J 6.0 (National Institutes of Health, Bethesda, MD, USA).

### Statistical Analysis

All data are expressed as the mean ± SD and analyzed using the software GraphPad Prism 8.0 (GraphPad Software, Inc., La Jolla, CA, USA) and SPSS 13.0 (SPSS, Inc., Chicago, IL, USA). The Wilcoxon rank sum test was used to compare two independent samples of the original data, and the Kruskal-Wallis test was used for non-normal distribution data. Multiple-group comparisons were evaluated by one-way ANOVA with post hoc testing. Values of *p* < 0.05 were considered statistically significant.

## Results

### The PCOS Model Was Successfully Established

**Fig. 1:**
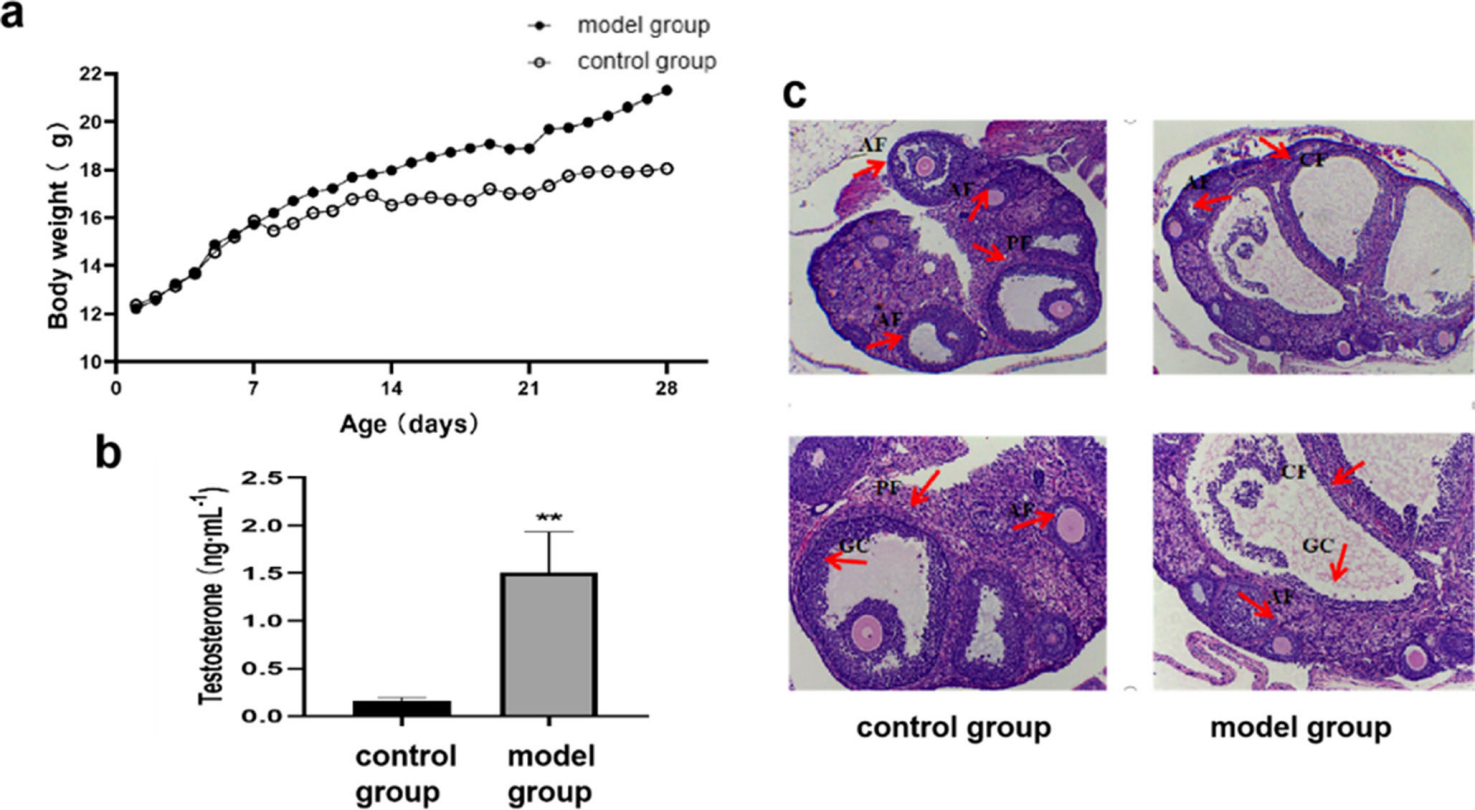
Effects of high-fat diet combined with DHEA on mice. **a** Mean of daily weight changes in control and PCOS mice (control group, *n* = 13; PCOS group, *n* = 53). **b** Difference of serum T between control group and PCOS model group (*n* = 3). **c** Ovarian tissue prepared from control group and PCOS group after H&E staining (*n* = 3). AF, antral follicle; PF, preovulatory follicle; CF, cystic follicle; GC, granulosa cell. Values are means ± SD. *p < 0.05, **p < 0.01 versus control group

As shown in Fig. 1a, the weight gain of mice in the model group was significantly different from that in the control group (*p* < 0.05). Figure 1b shows that the serum testosterone level in the PCOS model group was significantly higher than that in the control group (*p* < 0.01), which is in agreement with the symptoms of hyperandrogenemia in PCOS. As shown in Fig. 1c, atresia and cystic dilated follicles were significantly increased in the model group compared with the control group. In addition, the number of granulosa cell layers was decreased, and the cells were loose. However, the ovaries of mice in the normal control group showed multiple follicles at different development stages. This indicates that the mouse PCOS model was successfully established.

### APN Increases the Pregnancy Rate of PCOS Mice and Reduces the Absorption Rate of Embryos

Since APN plays an important role in anti-apoptosis and improves the embryonic development, we studied the effect of APN on pregnancy rate and embryo loss rate in mice. The pregnancy rates of control, PCOS, and APN + PCOS groups were 77.78%, 35.29%, and 66.67%, respectively. As shown in Table 2, the embryonic absorption rate of the PCOS group was significantly higher than that of the control group (*χ*^2^ = 5.330, *p* < 0.05), while that of the APN + PCOS group was significantly lower than that of the PCOS group (*χ*^2^ = 4.623, *p* < 0.05). These findings demonstrate that abortion rate and embryo loss rate of PCOS model pregnant mice were significantly higher than those of the control group. APN increased the pregnancy rate of mice and reduced their embryo absorption rate, which resulted in an improved pregnancy outcome of PCOS model pregnant mice.

**Table 2:** Embryonic absorption rate in each group

Groups	Survived embryos (pcs)	Absorbed embryos (pcs)	Embryo absorption rate (%)	*χ*^2^ values	*p* values
Control group	90	29	24.4		
PCOS group	52	36	40.9	5.330	0.030*
APN + PCOS group	111	42	27.5	4.623	0.045^#^

*Compared with the control group, ^#^compared with the PCOS group

### Effects of APN on the Activities of AMPK, PI3K, and Akt In Vivo

**Fig. 2:**
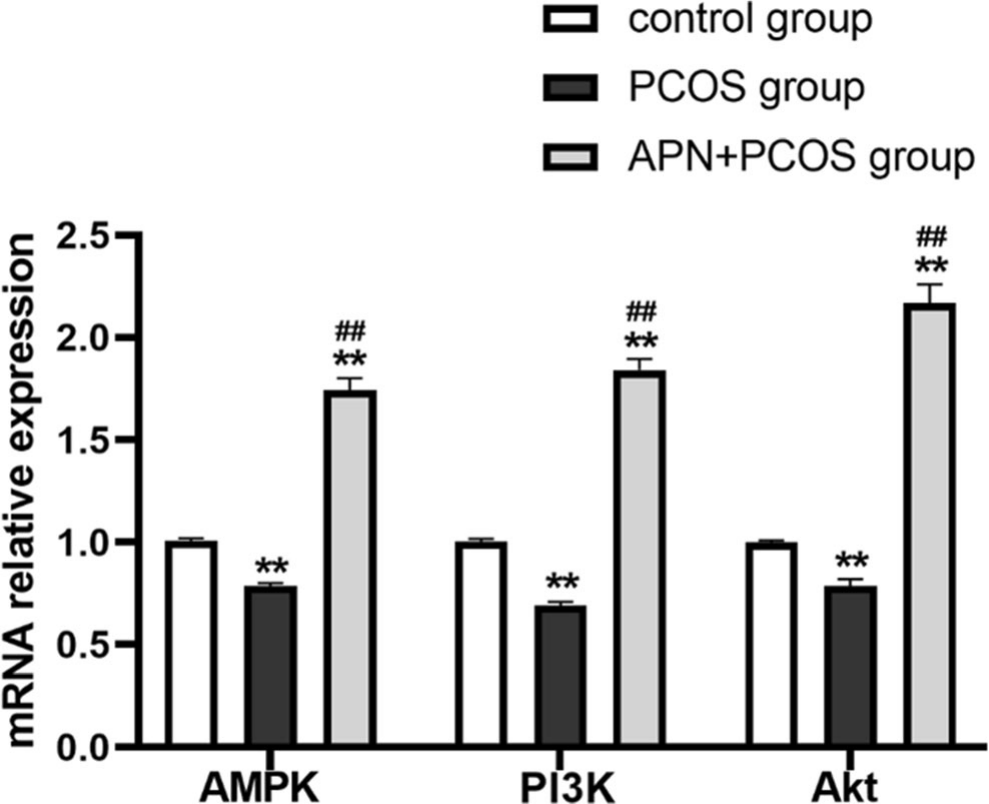
Relative expressions of AMPK, PI3K, and Akt mRNA in uterine tissues of mice in control, PCOS model, and APN + PCOS groups were compared. Relative expression is the average of 2^-ΔΔCt^ ± SD (*n* = 6). **p < 0.001 versus control group; ##*p* < 0.001 versus PCOS group

To further confirm that the effect of APN on the absorption rate of mouse embryos is related to AMPK, PI3K, and Akt, the mRNA expression levels of these factors were measured by qPCR. As shown in Fig. 2, the relative mRNA expressions of AMPK, PI3K, and Akt in the PCOS model group were decreased compared with the control group (*p* < 0.001). The mRNA expression levels of AMPK, PI3K, and Akt were all increased in the APN + PCOS group (*p* < 0.001). The relative mRNA expression levels of AMPK, PI3K, and Akt were significantly higher in the APN + PCOS group than in the PCOS model group (*p* < 0.001). The differences were statistically significant, and the mRNA expression levels of AMPK, PI3K, and Akt in uterine tissues of mice in the three groups showed the same trend.

**Fig. 3:**
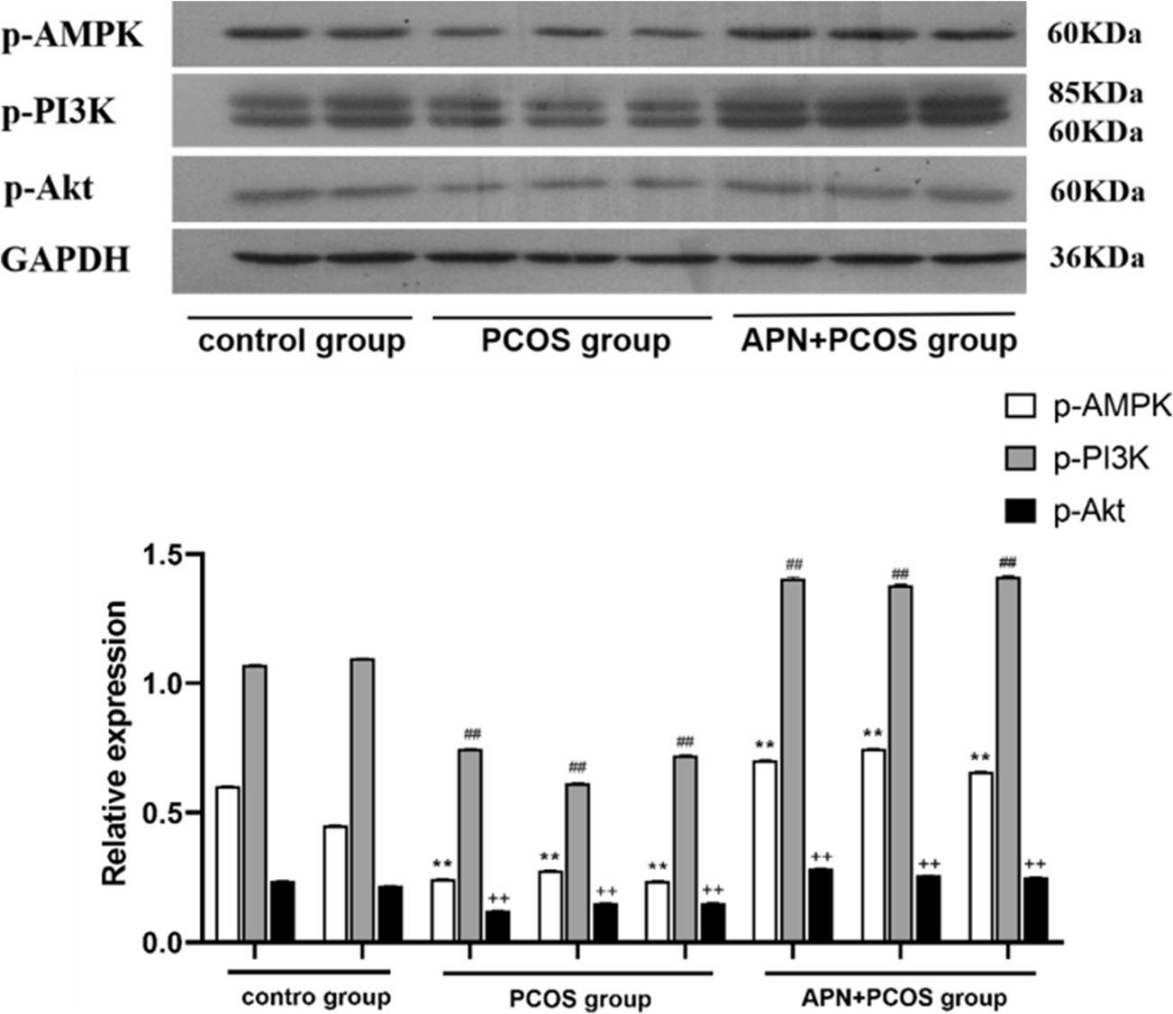
Protein expressions of p-AMPK, p-PI3K, and p-Akt in uterine tissues of mice in control, PCOS model, and APN + PCOS groups were compared. **p < 0.001 versus control group; ##*p* < 0.001 versus control group; ++*p* < 0.001 versus control group

To further clarify the effect of APN on the expression levels of AMPK, PI3K, and Akt in PCOS mouse uterine tissue, Western blot was used to analyze the protein expressions of phosphorylated AMPK (p-AMPK), phosphorylated PI3K (p-PI3K), and phosphorylated Akt (p-Akt). The results shown in Fig. 3 demonstrate that the protein expression levels of p-AMPK, p-PI3K, and p-Akt in the PCOS group were significantly decreased compared with the control group (*p* < 0.001). The expression levels of p-AMPK, p-PI3K, and p-Akt in the APN + PCOS group were significantly higher than those in the control group and the PCOS group (*p* < 0.001). This finding suggests that the protective effects of APN on PCOS mice in early pregnancy is at least partially due to enhanced phosphorylation of AMPK, PI3K, and Akt.

As expected, these results unveiled that APN increases the phosphorylation of AMPK, PI3K, and Akt as well as the mRNA expression levels of AMPK, PI3K, and Akt. These results indicate that exogenous APN may reduce the embryo loss rate in early pregnancy of PCOS mice by activating the AMPK/PI3K/Akt pathway.

### APN Ameliorates Apoptosis of Trophoblast Cells in PCOS Mice in High-Glucose Environment

**Fig. 4:**
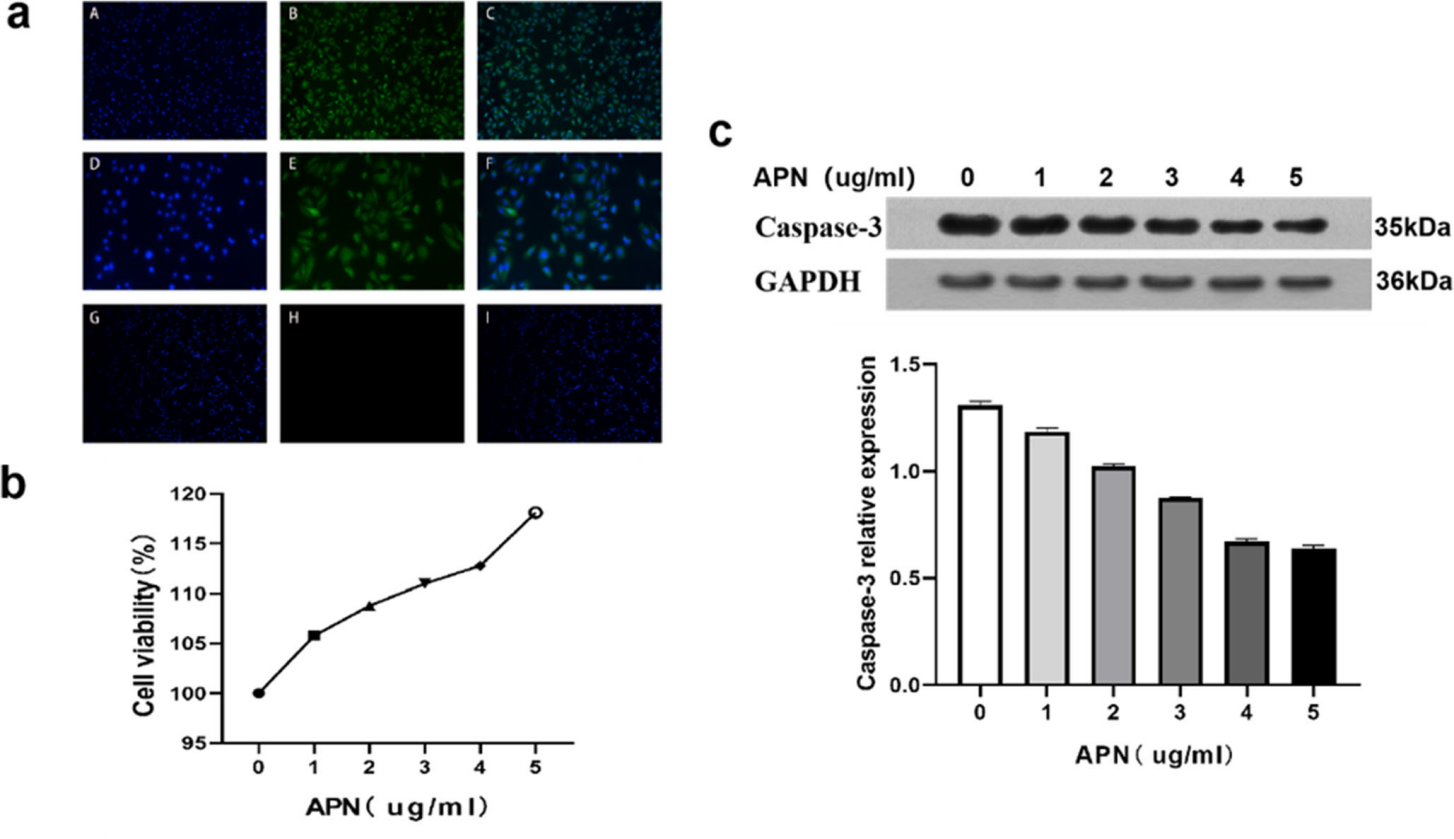
APN improves apoptosis of trophoblast cells of PCOS mice in the high-glucose environment. **a** Immunofluorescence identification of villous trophoblast cells in PCOS model mice. A–C were positive for CK18 (× 100): A, DAPI; B, fluorescence; and C, merge. D–F were positive for CK18 (× 200): D, DAPI; E, fluorescence; and F, merge. G–I were negative for CK18 (× 100): G, DAPI; H, fluorescence; and I, merge. **b** Relationship between the activity of trophoblast cells and APN concentration in a high-glucose environment. **c** Western blot was used to detect the effects of different APN concentrations on the relative expression levels of caspase-3 protein in each cell group in the high-glucose environment. The Glu concentration in the high-glucose environment was 30 mM, and the APN concentration was 1–5 μg/mL

Immunofluorescence showed that the cultured cells were villi trophoblast cells (Fig. 4a). The high-glucose environment induces the apoptosis of villi trophoblast cells in vitro. Therefore, the MTT method was used to detect the effects of different APN concentrations on the proliferation of trophoblast cells of PCOS mice in the high-glucose environment (Glu 30 mM), showing the highest cell viability for the APN concentration of 5 μg/mL (Fig. 4b). Therefore, this APN concentration was chosen for the following experiments.

Caspase-3 is an apoptosis-related factor that is widely used for the detection of apoptosis. The relative protein expression of caspase-3 is positively correlated with the degree of apoptosis of trophoblast cells. Western blot was used to detect the relative protein expression of caspase-3 in cell culture supernatant and to observe the changes of apoptosis by APN of different concentrations. The results suggest that APN improves apoptosis most significantly at the APN concentration of 5 μg/mL (Fig. 4c).

### APN Ameliorates Trophoblast Apoptosis by Activating the AMPK/PI3K/Akt/FoxO3a Pathway

**Fig. 5:**
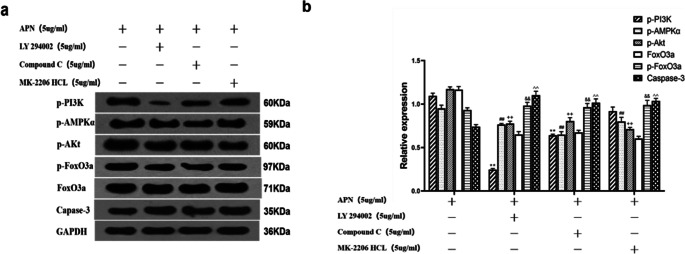
**a**, **b** APN improves the apoptosis of villi trophoblast cells in PCOS mice by acting on the AMPK/PI3K/Akt/FoxO3a pathway. Cultured trophoblast cells were treated with 5 μg/mL APN, 5 μg/mL Compound C, 5 μg/mL LY294002, or 5 μg/mL MK- 2206 HCL in a high-glucose environment (Glu 30 mM). Protein expressions of p-PI3K, p-AMPKα, p-AKT, FoxO3a, P–FoxO3a, and caspase-3 were measured by Western blot analysis. Data are expressed as mean ± SD. **p < 0.001 versus control group; ##*p* < 0.001 versus control group; ++*p* < 0.001 versus control group; ^&&^*p* < 0.001 versus control group; ^^*p* < 0.001 versus control group

To examine whether APN acts on the AMPK/PI3K/Akt/FoxO3a pathway, we divided trophoblast cells of mice cultured in a high-glucose environment (Glu 30 mM) into four groups: (A) without pretreatment, (B) with Compound C pretreatment, (C) with LY 294002 pretreatment, and (D) with MK–2206 HCL pretreatment. Compound C is a selective, ATP-competitive AMPK inhibitor used to inhibit AMPK and observe the phosphorylation of PI3K, Akt, and FoxO3a. LY 294002 is a PI3K inhibitor used to inhibit the action of PI3K and to observe the phosphorylation of AMPK, Akt, and FoxO3a. MK-2206 HCL is an Akt inhibitor. When Akt is inhibited, the phosphorylation of AMPK, PI3K, and FoxO3a are observed. All three inhibitors were used to test the relationship between AMPK, PI3K, and Akt and to verify the existence of AMPK/PI3K/Akt/FoxO3a pathway. After 48 h, the relative expressions of p-PI3K, p-AMPKα, p-AKT, FoxO3a, p-FoxO3a, and caspase-3 were detected by Western blot (Fig. 5a, b). PI3K, Akt, and FoxO3a phosphorylation were inhibited by pretreatment with the AMPK inhibitor Compound C. Moreover, AMPKα, Akt, and FoxO3a phosphorylation were inhibited by pretreatment with the PI3K inhibitor LY294002, and FoxO3a phosphorylation was inhibited by pretreatment with MK-2206 HCL. These results prove that the protein expression of PI3K/Akt is affected by AMPK and that AMPK interacts with the PI3K/Akt pathway. The relative expression of caspase-3 was increased when Compound C, LY 294002, or MK–2206 HCL were given, indicating that APN improves the apoptosis of trophoblast cells in PCOS mice by affecting AMPK, PI3K, Akt, and FoxO3a. These results reveal that APN ameliorates the apoptosis of villi trophoblast cells in PCOS mice by acting on the AMPK/PI3K/Akt/FoxO3a pathway.

## Discussion and Conclusions

PCOS is a complex metabolic syndrome and the principal ovarian factor that causes infertility in women of reproductive age [21]. Although the pregnancy rate of PCOS patients can be improved by various clinical treatments, the abortion rate in early pregnancy is still higher than that of normal pregnant women. Studies have shown that the serum APN level of PCOS pregnancy patients is significantly lower than that of normal pregnancy patients [22]. Furthermore, the expression of the endometrial APN receptor is significantly lower in patients with repeated embryo implantation failure for unknown reasons [23]. These findings suggest that an abnormal APN level may affect the outcome of pregnancy. However, the mechanism by which PCOS affects the pregnancy outcome is still unclear, and animal experiments gave no convincing evidence. We successfully established and applied the model to explore the correlation between the high abortion rate caused by PCOS and the imbalance of the APN level. The results showed that, compared with normal control mice, PCOS model mice had an increased embryo absorption rate but a decreased pregnancy rate. This indicates that APN increases the pregnancy rate of PCOS mice, reduces the embryonic absorption rate, and improves the pregnancy outcome of PCOS model pregnant mice. These effects may be related to the direct regulation of reproduction and embryo implantation by APN in the embryonic development as well as the ability to promote the synthesis and invasion of trophoblast cells in early pregnancy [18, 24].

In order to further explore the possible mechanism of APN action in vivo, we injected exogenous APN subcutaneously in PCOS model mice and measured the relative expressions of AMPK, PI3K, and Akt proteins as well as mRNA in uterine tissues. We found that the phosphorylation levels of AMPK, PI3K, and Akt were up-regulated in the uterine tissue of the model mice, which indicates that the protective effect of APN on PCOS mouse embryos in early pregnancy is at least partly due to increased phosphorylation of AMPK, PI3K, and Akt. AMPK/PI3K/Akt has been demonstrated to be a key coordinator in protecting cells against oxidative and inflammatory insults [25]. AMPK is a serine/threonine kinase and a sensor and regulator of the cellular energy metabolism. When activated, it increases the cell’s AMP/ATP ratio, leading to phosphorylation [26]. AMPK and PI3K/Akt pathways are not isolated from each other but have complex interactions. After activation of AMPK, adverse pregnancy outcomes due to insulin resistance can be reversed by an interaction between AMPK and the PI3K/Akt signaling pathway [27]. Activated AMPK triggers a series of reactions that reduce androgen production and insulin resistance. This reduction promotes ovulation, which is critical to the success of embryo implantation, and increases pregnancy rates. Therefore, activation of AMPK can reverse the adverse pregnancy outcome caused by insulin resistance through the interaction between AMPK and the PI3K/Akt signaling pathway [28]. However, the researchers also had some inconsistent results; some researchers shown that plasma APN levels are significantly higher in women with recurrent abortions [29]. This finding may be related to the proinflammatory role of APN in human placental tissue, as APN stimulates the production of proinflammatory cytokines and prostaglandins through trophoblast cells and inhibits the supply of placental glucose and amino acids to play a pro-apoptotic role [28].

To further investigate how APN improves embryonic development and trophoblast apoptosis by affecting AMPK, PI3K, and Akt expression and the interaction between these pathways, villi trophoblast cells of PCOS model mice were cultured in vitro. APN, AMPK inhibitor Compound C, PI3K inhibitor LY 294002, or Akt inhibitor MK–2206 HCL were individually given in high-glucose environment, and the relative expressions of the corresponding proteins were detected by Western blot. As expected, APN significantly improves embryo loss rate and trophoblast apoptosis in PCOS model mice, and the potential underlying molecular mechanism is related to the AMPK/PI3K/Akt/FoxO3a signaling pathway. When Compound C was given, the relative expressions of p-PI3K, p-Akt, and p-FoxO3a were decreased compared with those of PCOS model mice that received APN alone. In contrast, the expression of caspase-3 was increased, indicating that APN enhances the expression of PI3K, Akt, and FoxO3a downstream by increasing the phosphorylation of AMPK, thereby ameliorating trophoblast apoptosis. Furthermore, the protein expression of PI3K/Akt can be affected by AMPK, which interacts with the PI3K/Akt pathway. When LY 294002 or MK–2206 HCL was given, phosphorylation of AMPK decreased in addition to the decrease in phosphorylation of Akt and FoxO3a downstream of AMPK. Furthermore, the relative expression of caspase-3 increased. This suggests that Akt regulates AMPK activity by phosphorylation of AMPK and that AMPK and the PI3K/Akt pathway are not isolated from each other but have complex interactions. However, some researchers have suggested that AMPK activation can promote phosphorylation of IRS-1 at Ser 794, thereby inhibiting the PI3K/Akt signaling pathway [30]. PI3K is reported to be an intracellular phosphatidylinositol kinase that is associated with various cellular functions, including cell proliferation, differentiation, apoptosis, and glucose transport [31]. The Akt signaling pathway is mainly responsible for the transmission of biological information initiated by PI3K. As a key downstream effector of PI3K, it plays an important role in various biological processes, including cell metabolism, cell cycle regulation, cell growth, and apoptosis through phosphorylation of various target proteins and downstream pathways [32]. Phosphorylation at Ser 485 in the AMPKα subunit is involved in the regulation of AMPK activity, and Ser 485 is one of the targets of Akt, so Akt regulates the activity of AMPK by phosphorylation at Ser 485 [33]. APN can activate the intracellular PI3K/Akt signaling pathway by binding to receptors, and FoxO3a protein is a direct target molecule of the PI3K/Akt signaling pathway. The activity of FoxO3a protein is mainly regulated by this pathway and closely related to the transcription of many downstream regulatory factors. The PI3K/Akt signaling pathway is believed to play an important regulatory role in energy metabolism and the reproductive ability of mammals [17]. Therefore, activation of the PI3K/Akt signaling pathway can promote trophoblast cell proliferation and inhibit apoptosis by affecting the FoxO3a protein. When the PI3K/Akt pathway is inhibited, dephosphorylated FoxO3a can induce apoptosis of oocytes by activating the apoptotic protein caspase-3 by the apoptotic factor [34].

In conclusion, this study proves that APN has good therapeutic potential in improving the adverse pregnancy outcome of PCOS due to its anti-apoptotic effect. Pregnancy loss in PCOS mice was prevented by activation of the AMPK/PI3K/Akt/FoxO3a signaling pathway. Due to its intricate endocrine and metabolic abnormalities, PCOS is closely related to abortion. However, the specific mechanism is still unclear and needs further exploration. Therefore, further study of this signaling pathway will not only help to understand the pathogenesis of PCOS but also further explore the relationship between PCOS and abortion and provide a new research idea for the treatment of PCOS as well as miscarriage caused by PCOS. We will continue to carry out further research along these lines in the future. With the continuous efforts and investment in this important area, more and more PCOS patients are expected to benefit from relevant treatment in the near future.

## Appendix

1. Adiponectin and its mechanism: Adiponectin (APN) is a protein of 244 amino acid residues encoded by apM1 gene and secreted specifically by fat cells. It is an important messenger for fat tissues to communicate with other organs [29, 35]. APN receptor genes (AdipoR1 and AdipoR2) are highly expressed in the secretory stage of the female endometrium, and the mRNA levels of APN, AdipoR1, and AdipoR2 are significantly increased in implantation, suggesting that APN is involved in the changes of the endometrium before implantation [36].
2. Mechanisms of AMPK, PI3K, Akt, and FoxO3a: Activation of AMPK inhibits phosphorylation of insulin receptor substrates 1 (Ser 636/639), thereby activating the intracellular insulin/phosphatidylinositol-3 kinase/protein kinase B (insulin /PI3K/Akt) signaling pathway [37]. PI3K is a kind of kinase that specifically catalyzes phosphatidylinositol and acts as lipid activator enzyme and serine/threonine protein kinase. Activated PI3K regulates the metabolism by activating or inhibiting its downstream target protein through phosphorylation. Akt is a member of the serine/threonine protein kinase family and a direct downstream target protein of PI3K. FoxO3a is a member of the fork box protein O (FOXO) family. Its main biological activity presents the binding with corresponding DNA, protein kinases, or transcription factors, regulating the downstream biomolecules through a series of pathways.
